# Integrative transcriptomic and TMT-based proteomic analysis reveals the mechanism by which *AtENO2* affects seed germination under salt stress

**DOI:** 10.3389/fpls.2022.1035750

**Published:** 2022-10-21

**Authors:** Yu Wu, Huimin Liu, Jie Bing, Genfa Zhang

**Affiliations:** Beijing Key Laboratory of Gene Resource and Molecular Development, College of Life Sciences, Beijing Normal University, Beijing, China

**Keywords:** *ENO2*, salt stress, seed germination, transcriptome, proteome, *Arabidopsis thaliana*

## Abstract

Seed germination is critical for plant survival and agricultural production and is affected by many cues, including internal factors and external environmental conditions. As a key enzyme in glycolysis, enolase 2 (ENO2) also plays a vital role in plant growth and abiotic stress responses. In our research, we found that the seed germination rate was lower in the *AtENO2* mutation (*eno2^-^*) than in the wild type (WT) under salt stress in *Arabidopsis thaliana*, while there was no significant difference under normal conditions. However, the mechanisms by which *AtENO2* regulates seed germination under salt stress remain limited. In the current study, transcriptome and proteome analyses were used to compare *eno2^-^* and the WT under normal and salt stress conditions at the germination stage. There were 417 and 4442 differentially expressed genes (DEGs) identified by transcriptome, and 302 and 1929 differentially expressed proteins (DEPs) qualified by proteome under normal and salt stress conditions, respectively. The combined analysis found abundant DEGs and DEPs related to stresses and hydrogen peroxide removal were highly down-regulated in *eno2^-^*. In addition, several DEGs and DEPs encoding phytohormone transduction pathways were identified, and the DEGs and DEPs related to ABA signaling were relatively greatly up-regulated in *eno2^-^*. Moreover, we constructed an interactive network and further identified GAPA1 and GAPB that could interact with AtENO2, which may explain the function of AtENO2 under salt stress during seed germination. Together, our results reveal that under salt stress, *AtENO2* mainly affects the expression of genes and proteins related to the phytohormone signal transduction pathways, stress response factors, and reactive oxygen species (ROS), and then affects seed germination. Our study lays the foundation for further exploration of the molecular function of *AtENO2* under salt stress at the seed germination stage in *Arabidopsis thaliana*.

## Introduction

The transition of seeds of higher plants from dormancy to germination is one of the key stages at the beginning of a new life cycle, which is of great significance for plants to complete their entire life cycle (Wu et al., 2021). Physiologically, seed germination is defined as the transformation of seeds from a relatively quiescent state to a vigorous stage. Morphologically, it shows that the radicle and germ break through the seed coat and elongate outward to develop into a new individual (Koller et al., 1962). Whether the seed can germinate smoothly depends on its internal factors, such as whether it has a complete embryo, sufficient nutrients, and the hormone content is balanced, which affects the germination of seeds. Abscisic acid (ABA) and gibberellin (GA) play indispensable roles. ABA mainly inhibits germination (Sun et al., 2021). In ABA-deficient mutants, ABA synthesis is inhibited, and the germination rate of the mutants is higher than that of the wild type (WT). In ABA overexpressing transgenic materials, the phenotype is the opposite (Martinez-Andujar et al., 2011; Nonogaki et al., 2014). A major mechanism by which GA promotes seed germination is by promoting the degradation of the DELLA protein RGA-LIKE 2 (RGL2), a major repressor of germination in *Arabidopsis* seeds (Lee et al., 2022). Meanwhile, the germination process is also affected by external environmental factors. As one of the main abiotic stresses affecting plant growth and development, salt stress also affects the germination of seeds. At present, the physiological effect of salt stress on seed germination is very clear, but different scholars hold different views on its specific mechanisms. Some studies have believed that the decrease in the germination rate under salt stress is mainly due to the high osmotic pressure caused by high salt, which makes the metabolic activities of seeds unable to be carried out due to insufficient water absorption, which affects germination (Munns and Tester, 2008). Some researchers thought that the effect of high salt on seed germination was mainly due to ion toxicity (Zhu et al., 2017; Liu et al., 2021). Based on previous studies, other studies suggested that high salt stress mainly affected the activity of amylase, leading to the obstruction of the process of providing energy and nutrients for radicles (Zhu et al., 2021).

With the continuous development of the research field, an increasing number of gene functions related to seed germination under salt stress have been identified. The *ENO* genes are widely distributed and highly conserved in mammals and plants and encode the glycolytic enzyme ENO2, which catalyzes the dehydration of 2-phospho-D-glycerate (2-PGA) to phosphoenolpyruvate (PEP) (Wu et al., 2021). In *Arabidopsis*, ENO1, ENO2, and ENO3 are the main isozymes, and the expression of ENO2 is much higher than that of ENO1 and ENO3 (Andriotis et al., 2010). More and more studies have shown that ENO2 is a multifunctional enzyme that not only plays a role in glycolysis but also plays an essential role in the normal growth and development of plants and response to abiotic stresses (Lee et al., 2002a; Kang et al., 2013; Eremina et al., 2015; Yang et al., 2021a). Our laboratory obtained homozygous mutants (*eno2^-^*) of *AtENO2* through continuous screening and identification. The absence of *AtENO2* causes defective growth and reproduction, such as stunted plants, a reduced germination rate of pollen, impaired floral organogenesis, and damaged male gametophytes (Eremina et al., 2015). In addition, the germination rate between *eno2^-^* and the WT was not significantly different on Murashige and Skoog (MS) medium, but under salt stress, the germination rate of the mutant was lower than that of the WT. However, the mechanisms of *AtENO2* affecting seed germination under salt stress are not clear thus far.

Recently, large-scale omics methods, such as genomics, metabolomics, transcriptomics, and proteomics, have made it possible to investigate the mechanisms of plant defenses against different abiotic stresses (Deshmukh et al., 2014; Calhoun et al., 2021; Zeng et al., 2021). Transcriptomics can comprehensively and quickly obtain almost all mRNA sequence information of a specific sample in a specific period and is widely used in basic research, clinical diagnosis, pharmaceutical studies, and other fields. Protein is the embodiment of biological function; therefore, the study of biological function is inseparable from proteomics. Several studies have been conducted to explore the salt stress response mechanisms in plant species, such as rice (*Oryza sativa*) (Zhao et al., 2021b), maize (*Zea mays*) (Wang et al., 2016), soybeans (*Glycine max*) (Arai et al., 2008), and other species. Numerous salt-responsive genes and proteins have been identified. To determine the exact mechanism by which *AtENO2* affects seed germination under salt stress in *Arabidopsis*, we considered the use of omics methods. To avoid the limitations of single omics, we employed a conjoint analysis. Transcriptomics and proteomics offer an effective approach for identifying genes, proteins, gene ontology (GO) terms, and associated pathways that are crucial for understanding the functions of *AtENO2* in seed germination under salt stress.

In our research, we aimed to identify DEGs and DEPs in *eno2^-^* under salt stress compared to the WT and to investigate the molecular mechanisms regulating seed germination under salinity. For this reason, we performed RNA-Seq and TMT with the WT and *eno2^-^* on MS medium with or without 100 mM NaCl. First, the germination phenotypes under normal and salt stress were counted, and the related physiological indices were detected. Finally, with the help of omics, we found 4249 DEGs and 1831 DEPs only under salt stress. Through joint analysis, the key genes, proteins, GO terms, and pathways were also identified in *eno2^-^* compared to WT. These results will provide more information for understanding the function of *ENO2* in seed germination in *Arabidopsis thaliana*.

## Result

### *AtENO2* regulates seed germination in response to abiotic stress

**Figure 1:**
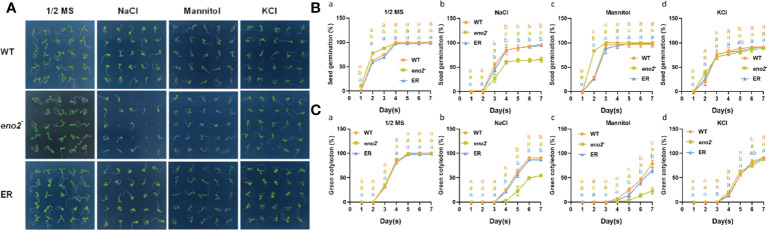
*AtENO2* regulates seed germination under salt stress. Seed germination of the WT, *eno2^-^*, and *ER* lines. Seeds were horizontally germinated on 1/2 MS medium with or without 100 mM NaCl, 200 mM mannitol, and 100 mM KCl for 7 days. Photographs were taken on the seventh day of germination **(A)**. The seed germination rate was measured at the indicated time point **B(a-d)**. The green cotyledon rate was measured every day for seven days **C(a-d)**. The orange, green, and blue dashed lines represent the WT, *eno2^-^*, and *ER*, respectively. Values are expressed as the means ± SD (n=3 replicates). Different letters indicate significant differences by one-way ANOVA (P < 0.05) with SPSS.

We set different concentrations of NaCl to explore the optimal concentration affecting seed germination, and finally, we chose 100 mM with an obvious difference for subsequent experiments (**Supplementary Figure S1**). After statistical analysis of the 1/2 MS medium, we found that *eno2^-^* was faster than the WT and *ER* in the first three days of germination, but there was no significant difference in the later stage of germination. On medium with NaCl, the seed germination of *eno2^-^* was more sensitive than that of the WT and *ER*. To investigate whether *ENO2* mainly affected *Arabidopsis* germination through ion toxicity or osmotic stress, we also investigated the germination of each line under isotonic conditions and K^+^ stress at the same concentration as Na^+^. On medium with mannitol, the seed germination rate showed no obvious difference among the genotypes. However, the green cotyledon percentage of the mutant was lower than that of the WT and *ER*. Additionally, on the medium with KCl, both the seed germination and green cotyledon rate displayed no significant difference among the lines (**Figure 1**). The leaf size and chlorophyll content during germination also showed similar growth tendencies (**Supplementary Figure S2**). These results indicated that under salt stress, *ENO2* mainly affected the content of Na^+^ or the cell signaling related to Na^+^ during seed germination, and had a certain degree of osmotic stress effect.

### Identification and classification of the DEGs between *eno2^-^* and WT under salt stress

Transcriptome sequencing was applied to reveal the effect of *ENO2* mutation on seed germination under normal and salt stress conditions. After analysis, we preliminarily determined the quality of RNA used for sequencing (**Supplementary Figure S3**, **Table S1**), and analyzed the base quality value (Q) and the Pearson correlation coefficient (R), etc (**Supplementary Figures S4**, **S5**, **Tables S1**, **S2**). All these data further indicated that our data met the sequencing requirements and could be used for further analysis.

**Figure 2:**
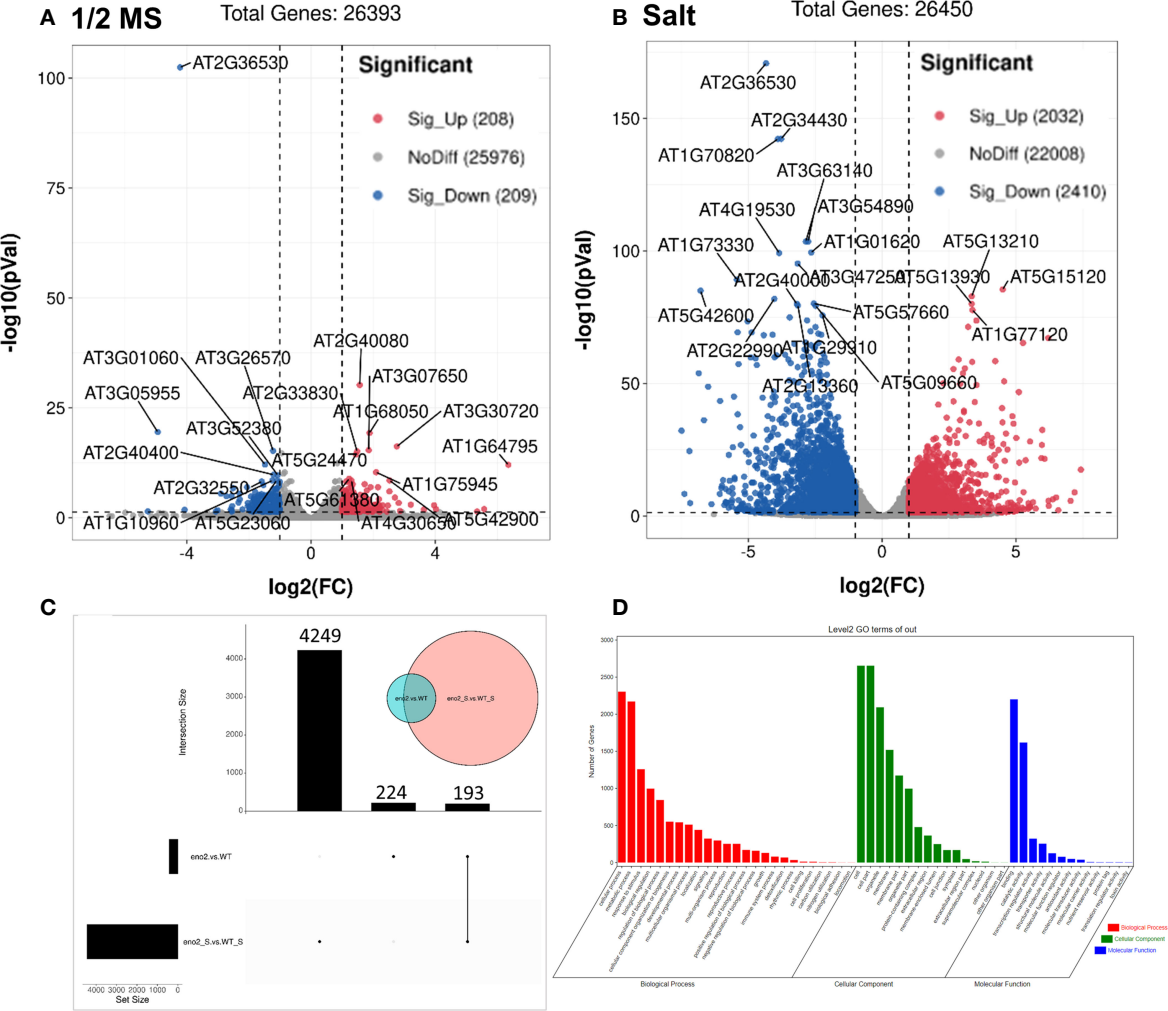
The analysis of DEGs in *eno2^-^* compared to the WT under different conditions. Volcano plot of the distribution of DEGs under normal conditions **(A)** and salt stress **(B)**. The X-axis represents the change in gene expression in different samples. The Y-axis indicates the statistical significance of changes in gene expression levels. The dots represent different genes. The grey dots represent genes with no significant difference, the red dots represent genes with significant differences in upregulation, and the blue dots represent genes with significant differences in downregulation. Transcriptomics UpSet diagram **(C)**. Green circles indicate genes that are differentially expressed under normal conditions, and pink circles indicate genes that are differentially expressed under salt stress. The left bar chart shows the number of DEGs under normal conditions. The middle column chart represents the number of DEGs under salt stress. And the right one means the DEGs that are common under normal and salt stress conditions. GO of key DEGs of *eno2^-^* compared to the WT under salt stress **(D)**. Red represents the classification of biological processes, green represents the classification of cellular components, and blue represents the classification of molecular functions.

To explore which genes would be affected by *ENO2* under salt stress, we analyzed the DEGs. A total of 417 DEGs were identified under normal conditions, of which 208 and 209 genes were upregulated and downregulated in *eno2^-^* plants, respectively (**Figure 2A**). Under salt stress, there were a total of 4442 significant DEGs, of which 2032 genes were significantly upregulated and 2410 genes were significantly downregulated (**Figure 2B**). To further identify the genes that were significantly expressed only under salt stress, the UpSet graph was drawn with the help of the Omicstudio cloud platform (https://www.omicstudio.cn/tool/43). A total of 193 genes were expressed jointly, 224 genes were expressed only under normal conditions, and 4249 genes were specifically expressed differently only under salt stress (**Figure 2C**). To further explore the functions of DEGs under salt stress, according to the previous analysis, we selected the 4249 genes that were significantly expressed only under salt stress for GO analysis. In addition, we used the OmicShare cloud platform to calculate the classification statistics (**Figure 2D**). The results showed that there were 54 GO terms, including 25 biological processes, 13 molecular functions, and 16 cellular components, with FDR<0.05 (**Table S4**). Of them, the top 3 terms were “cellular process” (2304 DEGs), “metabolic process” (2171 DEGs), and “response to stimulus” (1258 DEGs) in the biological process category. The GO terms with the highest DEG count number in the cellular component category were “cell” and “cell part” (both have 2654 DEGs), followed by “organelle” (2093 DEGs) and “membrane” (1519 DEGs). Molecular functions such as “binding” (2200 DEGs), “catalytic activity” (1618 DEGs), and “transcription regulator activity” (322 DEGs) were significantly enriched.

### Identification and classification of the DEPs between *eno2^-^* and WT under salt stress

Although transcriptome data can provide abundant information at the transcriptional level, they only represent the intermediate state of gene expression and only represent the potential protein expression and functional significance. Proteins are the real functional embodiment of organisms. Therefore, to obtain the difference in protein expression levels in comparison groups, TMT quantitative proteomics was used in our study. The quality of the samples sent for sequencing was tested (**Supplementary Figure S6**; **Table S5**), and there was good repeatability in the data groups and great differences between groups (**Supplementary Figures S7**, **S8**), which could be used for further analysis.

**Figure 3:**
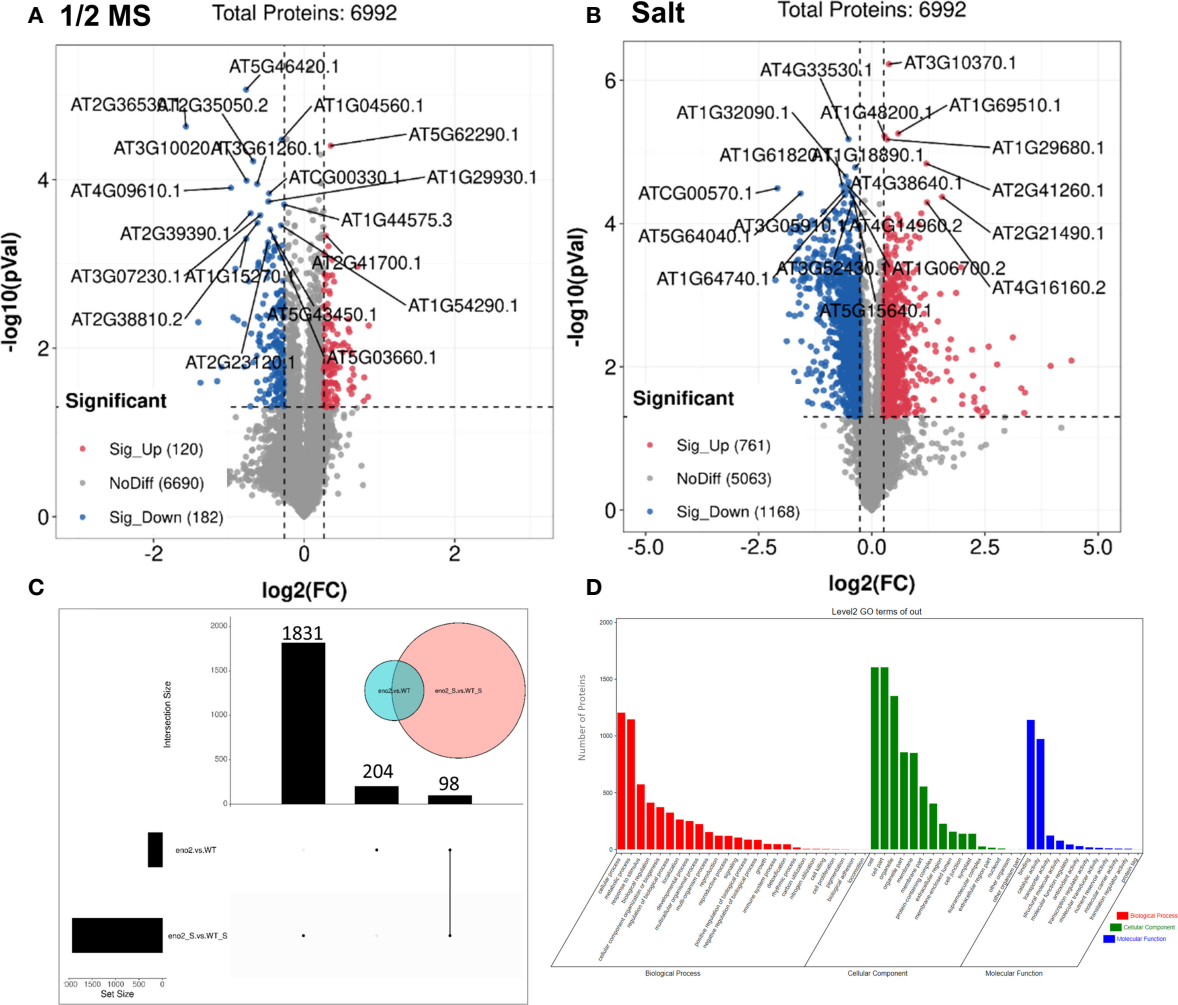
The analysis of differentially expressed proteins (DEPs) in *eno2^-^* compared to the WT under different conditions.Volcano plot of the distribution of DEPs under normal conditions **(A)** and salt stress **(B)**. The X-axis is the multiple of difference (logarithmic transformation based on 2), the Y-axis is the significance p-value of the difference (logarithmic transformation based on 10), the red dots are the upregulated significance differentially expressed proteins, the blue dots are downregulated significance differentially expressed proteins, and the grey dots are the proteins with no difference. Proteomics UpSet diagram **(C)**. Green circles indicate proteins that are differentially expressed under normal conditions, and pink circles indicate proteins that are differentially expressed under salt stress. The left bar chart shows the number of DEGs under normal conditions. The middle column chart represents the number of DEGs under salt stress. And the right one means the DEGs that are common under normal and salt stress conditions. GO of key DEPs of *eno2^-^* compared to the WT under salt stress **(D)**. Red represents the classification of biological processes, green represents the classification of cellular components, and blue represents the classification of molecular functions.

To analyze proteins with different expression differences between different groups, the DEPs were further screened. We found that under normal growth conditions, when *eno2^-^* was compared with the WT, a total of 302 proteins changed significantly, including 120 upregulated proteins and 182 downregulated proteins (**Figure 3A**). However, under salt stress, a total of 1929 proteins of *eno2^-^* were significantly different from the WT, including 761 significantly upregulated proteins and 1168 significantly downregulated proteins (**Figure 3B**). To further identify the proteins that were significantly expressed only under salt stress, the UpSet graph was drawn with the Omicstudio cloud platform, from which 98 proteins were expressed jointly, 204 proteins were expressed only under normal conditions, and 1831 proteins were specifically expressed differently only under salt stress (**Figure 3C**). To fully understand the function, localization, and biological pathway of proteins in organisms, proteins were annotated through GO. We used the OmicShare cloud platform to perform GO annotation analysis on 1831 proteins that were significantly differentially expressed only under salt stress after the above analysis (**Figure 3D**). The results showed that there were 54 GO terms, including 26 biological processes, 12 molecular functions, and 16 cellular components, with FDR<0.05 (**Table S6**). The top 3 terms were “cellular process” (1205 DEPs), “metabolic process” (1146 DEPs), and “response to stimulus” (573 DEPs) in the terms of biological process, which were the same as RNA-Seq. The GO terms with the highest number of DEPs in the cellular component category were “cell” and “cell part” (both had 1605 DEPs). In addition, “binding” (1141 DEPs), “catalytic activity” (973 DEPs), and “transporter activity” (122 DEPs) were significantly enriched in molecular function.

### Conjoint analysis of transcriptome and proteome data

**Figure 4:**
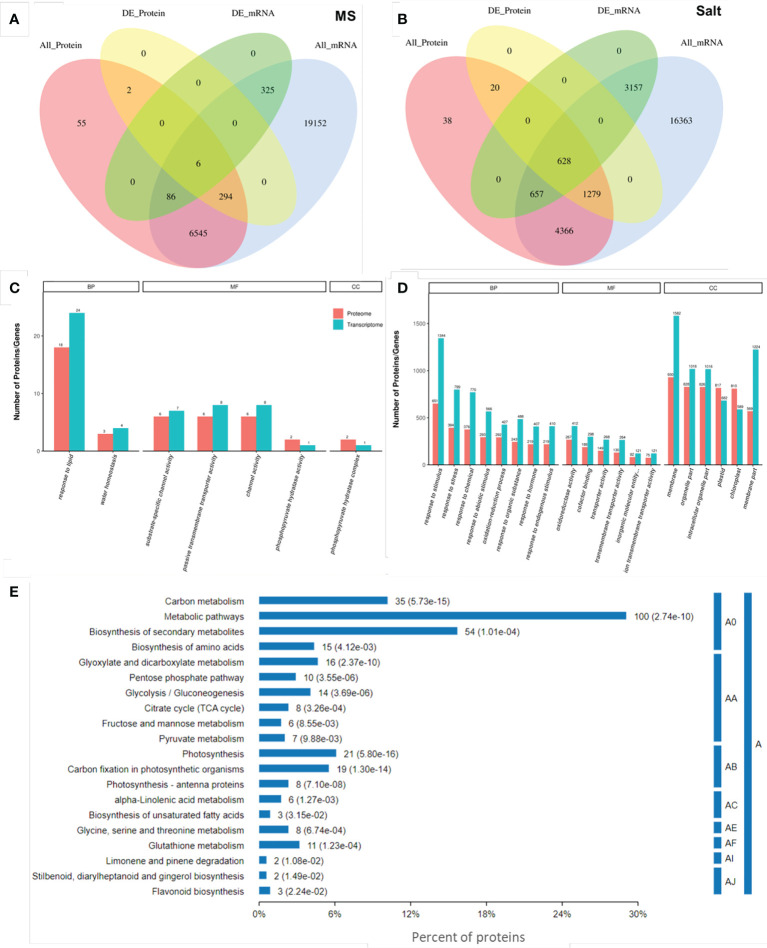
Combined analysis of transcriptome and proteome. Quantitative Venn diagrams of the transcriptome and proteome at the quantitative and differential expression levels under normal **(A)** and salt stress **(B)**. All_protein represents all quantifiable proteins in the proteome. All_Gene represents all quantifiable genes obtained from the transcriptome. DE_Protein indicates the differential proteins identified by the proteome, and DE_Gene is the differentially expressed gene that represents transcriptome identification. GO items are enriched under normal **(C)** and salt stress conditions **(D)**. Each column represents a GO second-level annotation item, red represents DEPs, and green represents DEGs. In the order of the number of DEPs contained from left to right, the higher the column is, the more active the GO second-level annotation item is in the tested samples. BP: Biological Process, MF: Molecular Function, and CC: Cellular Component. **(E)** KEGG pathway analysis of the DEPs in association under salt stress. A: Metabolism, A0: global and overview maps, AA: carbohydrate metabolism, AB: energy metabolism, AC: lipid metabolism, AE: amino acid metabolism, AF: metabolism of other amino acids, AI: metabolism of terpenoids and polyketides, and AJ: biosynthesis of other secondary metabolites.

Generally, biological phenomena are complex and variable, and internal regulation is complex; therefore, the conclusion of a single omics study is often not comprehensive. Therefore, we conducted a joint analysis of transcriptome and proteomic sequencing, hoping to have a more systematic exploration of the sequencing data. We integrated quantitative and differential expression information of the transcriptome and proteome conducted quantitative correlation analysis and drew Venn diagrams. Under normal growth conditions, only 6 genes and proteins were associated (**Figure 4A**), while 628 were associated under salt stress (**Figure 4B**).

In addition, we mapped the GO annotation map of genes and proteins associated under normal and salt stress conditions, respectively. On the MS medium, in 2 biological processes, the DEPs and DEGs were mainly enriched in “response to lipid” (18 DEPs and 24 DEGs) and “water homeostasis” (3 DEPs and 4 DEGs). Among the 4 molecular functions, the DEPs and DEGs were enriched in “substrate-specific channel activity” (6 DEPs and 7 DEGs), “passive transmembrane transporter activity” (6 DEPs and 8 DEGs), “channel activity” (6 DEPs and 8 DEGs), and “phosphopyruvate hydratase activity” (2 DEPs and 1 DEGs). Only the “phosphopyruvate hydratase complex” (2 DEPs and 1 DEGs) was enriched in cell components (**Figure 4C**; **Table S7**). Under salt stress, response to stimulus (651 DEPs and 1344 DEGs), response to stress (394 DEPs and 799 DEGs), “response to abiotic stimulus” (293 DEPs and 566 DEGs), “response to hormone” (219 DEPs and 407 DEGs), and other GO terms were enriched in biological process. The top 3 terms were “oxidoreductase activity” (267 DEPs and 412 DEGs), “cofactor binding” (188 DEPs and 298 DEGs), and “transporter activity” (149 DEPs and 268 DEGs). In addition, “membrane” (930 DEPs and 1582 DEGs), “organelle part” (828 DEPs and 1018 DEGs), and “intracellular organelle part” (826 DEPs and 1016 DEGs) were significantly enriched in cell components (**Figure 4D**; **Table S8**). These associated GO terms preliminarily indicated that *ENO2* plays a role mainly by affecting the expression of stress-related genes and proteins.

To further elucidate the pathway changes associated with DEPs under salt stress after combined analysis, proteins were annotated through the Kyoto Encyclopedia of Genes and Genomes (KEGG) pathway database. The KEGG analysis revealed that 74 pathways were mapped, and 20 pathways were significantly enriched (p-value <0.05) (**Figure 4E**). KEGG pathway enrichment analysis further found that “Metabolic pathways”, “Biosynthesis of secondary metabolites”, “Carbon metabolism”, “Photosynthesis”, “Carbon fixation in photosynthetic organisms”, and “Glycolysis/Gluconeogenesis” were significantly enriched. All these significantly enriched pathways were related to metabolism.

### DEPs and DEGs associated with stress in the combined analysis

**Figure 5:**
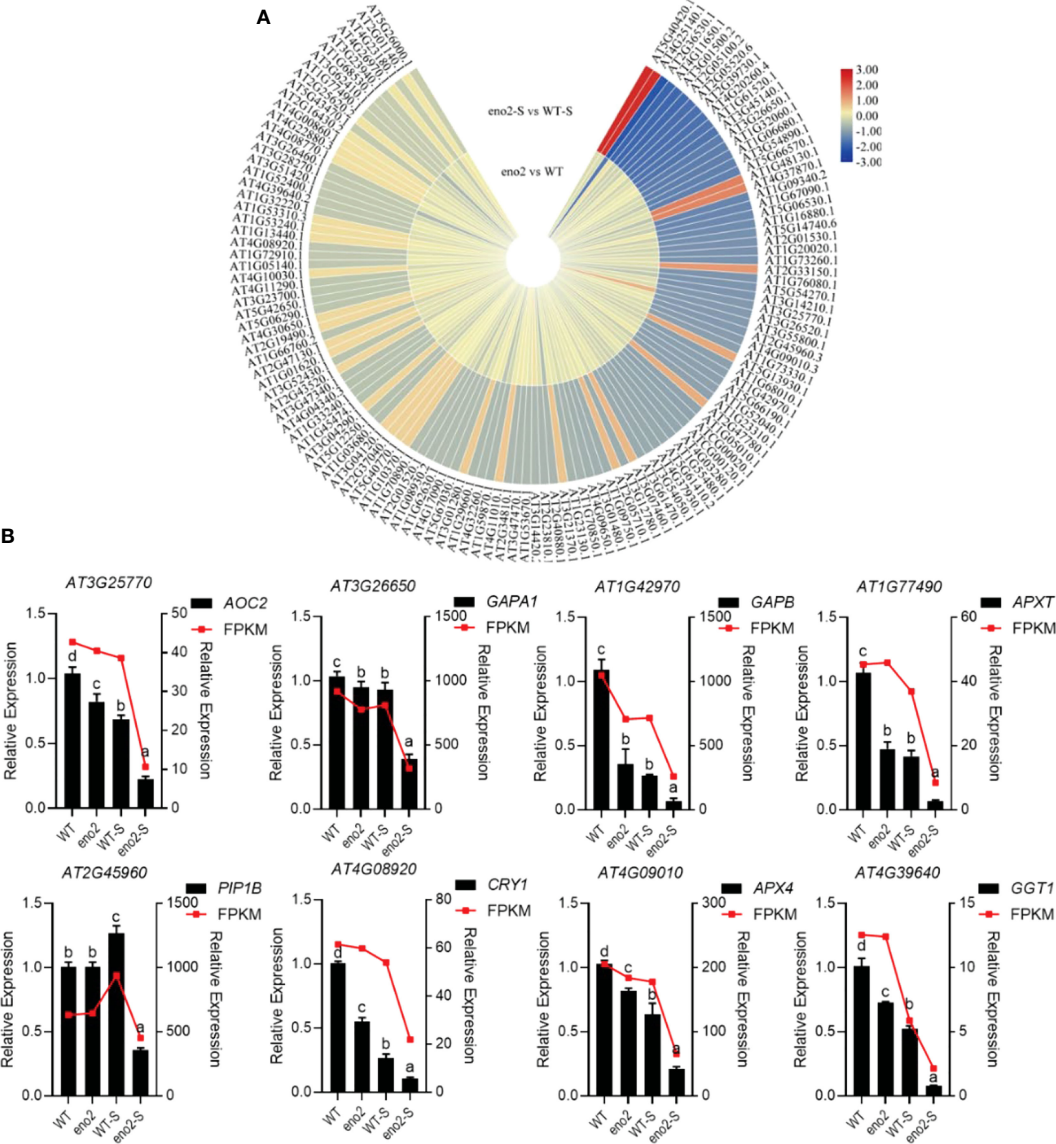
Heatmap of stress-related protein expression in the combined analysis and qRT–PCR validation of corresponding genes Expression of stress-related DEPs in the combined analysis **(A)**. The heatmap was drawn with log2FC by TBtools (Chen et al., 2020). The red to yellow colors represent significantly upregulated proteins, and the yellow to blue colors represent significantly downregulated proteins. qRT–PCR validation of DEGs that were related to stress **(B)**. The FPKM value was taken as the relative expression level of DEGs, which is shown as the red line, corresponding to the Y-axis on the right side. The qRT–PCR validation used the black bar chart, corresponding to the Y-axis on the left side, and *UBQ5* was used for each sample as an endogenous control. Three independent biologicals and three technical replicates were employed. Different letters indicate significant differences by one-way ANOVA (P < 0.05) with SPSS.

Seed germination can be affected by the external environment. Phenotypic analysis showed that under normal growth conditions, there was no significant difference between the WT and *eno2^-^* in germination; nevertheless, under a certain degree of salt stress, the germination rate and cotyledon- greening rate were significantly lower in *eno2^-^*. Consequently, we intended to explore which proteins and genes related to tolerance were mainly affected by *ENO2* under salt stress. The previous part revealed the changes in protein and gene levels caused by *ENO2* under normal and salt stress conditions from transcriptome and proteomics, respectively. Meanwhile, the sequencing data were further analyzed by joint analysis, which could avoid the limitation of relying only on a single omics analysis. To further investigate the results of the joint analysis, we first selected the stress-related proteins and genes significantly enriched in the GO annotation (**Figure 4D**). There were 799 DEGs enriched in the stress GO term in the transcriptome data, and 394 DEPs enriched in the stress GO term in proteomics. A total of 127 were significantly enriched in both the transcriptome and proteome (**Table S9**), in which the protein expression is shown in the form of a heatmap (**Figure 5A**).

In the process of plant roots absorbing water from the soil and transporting water to various tissues of plants, aquaporin-mediated water transport across membranes plays a crucial role (Maurel et al., 2015). *PIP1B* (*PIP1;2*) is a vital member of the *Arabidopsis* aquaporin family. Under normal conditions, there was no significant difference in the expression of PIP1B between the WT and *eno2^-^*, but it was significantly reduced under salt stress. Transcriptome and proteome data were maintained consistently, and the data reliability of the transcriptome was verified by qRT–PCR (**Figure 5B**). Peroxidases are important respiratory enzymes in plants. Their content is closely related to phenol metabolism and plant resistance (Gaudet et al., 2011). Generally, the higher its activity is, the stronger the stress resistance of the plant; in contrast, its resistance is reduced. L-ascorbate peroxidase T, chloroplastic (EC:1.11.1.11) (APXT) plays an important role in the scavenging process of reactive oxygen species. Under normal conditions, APXT in *eno2^-^* was lower than that in the WT. Under salt stress, APXT in the WT and *eno2^-^* was lower than that under normal conditions; in addition, APXT in mutants was significantly lower than that in the WT. In addition, under salt stress, the expression levels of cryptochrome-1 (CRY1), glyceraldehyde-3-phosphate dehydrogenase GAPA1, chloroplastic (EC:1.2.1.13) (GAPA1), glyceraldehyde-3-phosphate dehydrogenase GAPB, chloroplastic (EC:1.2.1.13) (GAPB), and other proteins in response to stress were significantly reduced in *eno2^-^*. The results of transcriptome sequencing were consistent with the expression trends of proteome sequencing. Meanwhile, some data were selected for qRT–PCR verification to further prove the reliability of the data.

### DEPs and DEGs associated with phytohormones in the combined analysis

**Figure 6:**
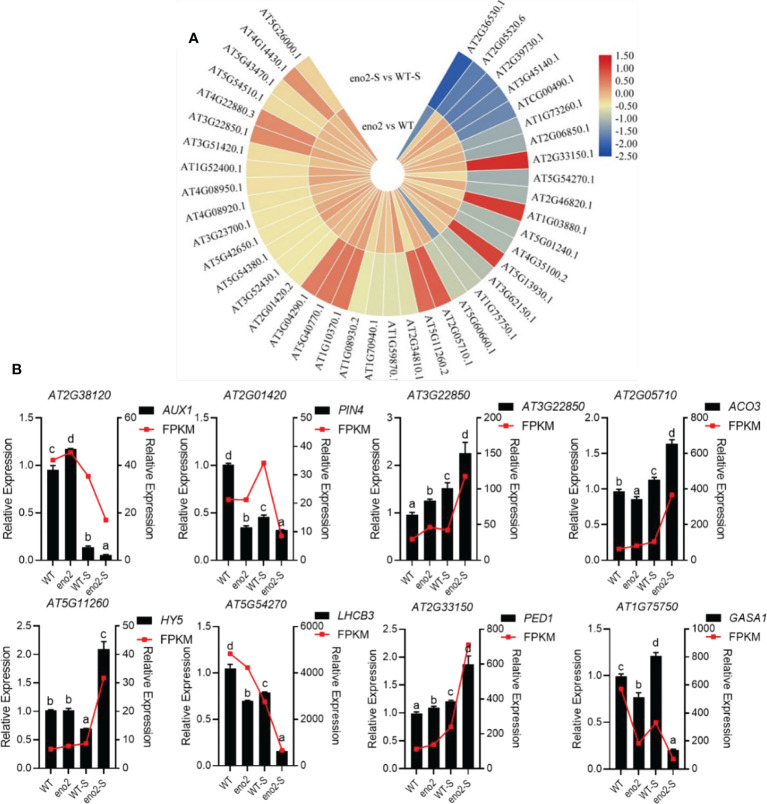
Heatmap of hormone-related protein expression in the combined analysis and qRT–PCR validation of corresponding genes. Expression of phytohormone-related DEPs in the combined analysis **(A)**. The heatmap was drawn with log2FC by TBtools (Chen et al., 2020). The red to yellow colors represent significantly upregulated proteins, and the yellow to blue colors represent significantly downregulated proteins. qRT–PCR validation of DEGs related to plant hormones **(B)**. The FPKM value was taken as the relative expression level of DEGs, which is shown as the red line, corresponding to the Y-axis on the right side. The qRT–PCR validation used the black bar chart, corresponding to the Y-axis on the left side, and *UBQ5* was used for each sample as an endogenous control. Three independent biologicals and three technical replicates were employed. Different letters indicate significant differences by one-way ANOVA (P < 0.05) with SPSS.

Phytohormones play indispensable roles in the process of seed germination. Plant endogenous hormones are produced under the action of external environmental signals. Through signal transduction, they induce the activation of enzymes and proteins and cause changes in cellular physiological metabolism. At present, GA, ABA, cytokinin (CK), and ethylene are the main endogenous hormones known to affect seed germination. To further investigate the results of the joint analysis, we selected phytohormone-related proteins and genes significantly enriched in the GO annotation (**Figure 4D**). There were 407 DEGs enriched in the “response to hormone” of transcriptome data, and 219 DEPs enriched in the phytohormone GO term in proteomics. A total of 41 were significantly enriched in both the transcriptome and proteome (**Table S10**), in which the protein expression is shown in the form of a heatmap (**Figure 6A**). At the same time, some data were randomly selected for qRT–PCR verification (**Figure 6B**), which further demonstrated the reliability of sequencing data.

In previous studies, ABA-insensitive *Arabidopsis* mutants and the WT were used for experiments. Under suitable germination conditions, the mutants could germinate directly without going through dormancy, indicating that ABA inhibited seed germination (Kermode, 2005; Song et al., 2020). Under salt stress, the expression levels of proteins related to the ABA-signaling pathway in *eno2^-^*, such as Aconitate hydratase 3, mitochondrial (EC:4.2.1.3) (ACO3), 3-ketoacyl-CoA thiolase 2, peroxisomal (EC:2.3.1.16) (PED1), and Basic-leucine zipper (BZIP) transcription factor family protein (HY5), were significantly upregulated, and the expression levels of genes were consistent with proteins. GA is a plant hormone closely related to seed germination and dormancy; however, its specific regulatory mechanism is not clear. Using GA alone did not improve seed germination. GA could help the radicle break through the limitation of the seed coat and promote seed germination by enhancing the growth ability of the embryo and softening the seed coat and endosperm (Yamauchi et al., 2004). Under salt stress, GA-regulated protein 1 (GASA1), which is related to GA synthesis, was significantly lower in *eno2^-^.* The content of auxin can also affect the seed germination process; usually, a low concentration (0.03~3 nmol/L) promotes seed germination (He et al., 2012), and a high concentration (0.3~1 μmol/L) inhibits germination (Liu et al., 2007). Under salt stress, the expression levels of genes and proteins related to the auxin-signaling pathway in *eno2^-^* were significantly lower than those in the WT. These changes in plant hormone content also explain the phenotype of seed germination in *eno2^-^*.

### Protein–protein interaction analysis and interaction protein validation

**Figure 7:**
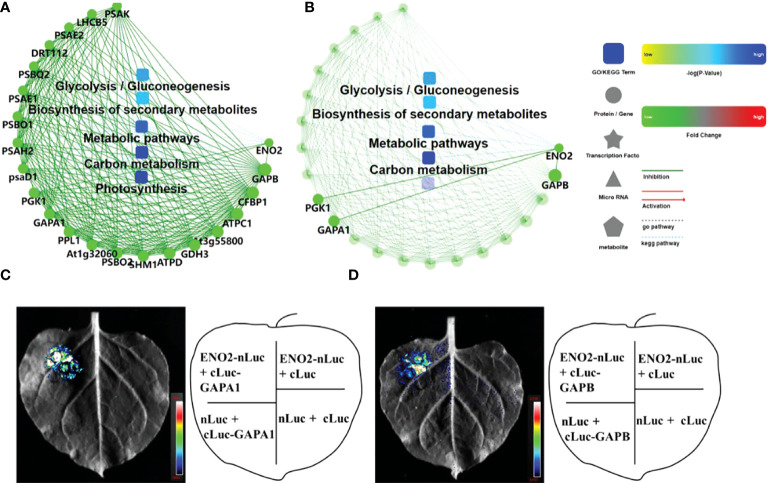
PPI network and interaction protein validation with LCA. **(A)** PPI of the proteins enriched in the top 5 KEGG pathways of Figure 5 **(E)**. **(B)** The proteins interacting with AtENO2 in **(A)**. Green represents downregulation, and red represents upregulation in *eno2^-^* compared to the WT. **(C)** Luciferase complementation assays (LCAs) revealed that ENO2 interacts with GAPA1. **(D)** LCA indicates that ENO2 interacts with GAPB. The pseudocolour represents the range of luminescence intensity in the image.

One of the important ways for proteins to perform their functions is to interact with other proteins and play biological regulatory roles through protein-mediated pathways or complex formation. Therefore, the study of protein–protein interactions is of great significance. In this study, OmicsBean was used to analyze the DEPs under salt stress after a combined analysis. The proteins from five pathways of the KEGG pathway (**Figure 4E**) were mainly selected to construct the PPI network (**Figure 7A**). In addition, we found that 3 proteins (PGK1, GAPA1, and GAPB) interacted with ENO2 (**Figure 7B**). Previous studies in our laboratory have proven that ENO2 can interact with PGK1 by Y2H (yeast two-hybrid) (Zhang et al., 2018). To verify PPI result, we next performed split luciferase complementation assays. Luciferase activity was detected in *Nicotiana benthamiana* leaves cotransformed with ENO2-nLuc and cLuc-GAPA1/GAPB constructs, whereas the negative control did not have signals, which indicates that ENO2 associates with GAPA1/GAPB *in vivo* (**Figures 7C, D**).

## Discussion

### *AtENO2* is a positive germination regulator under salt stress

In *Arabidopsis*, as a glycolytic metalloenzyme, AtENO2 catalyzes the dehydration of 2-phospho-D-glycerate (2-PGA) to phosphoenolpyruvate (PEP). Previous studies have shown that ENO2 is not only essential in glycolysis but also plays an indispensable role in the normal growth of *Arabidopsis* and response to abiotic stress. Twelve-day-old seedlings of *eno2^-^* had much shorter roots as well as smaller, pale green leaves, with shorter petioles than the WT. The mutations were characterized by severe dwarfism (Eremina et al., 2015). Our laboratory found that ENO2 can influence seed size and weight by adjusting the CK content and forming the ENO2-bZIP75 complex (Liu et al., 2020). *eno2^-^* impaired cold-responsive gene transcription, acquired freezing tolerance, and plant resistance to chilling under certain conditions (Lee et al., 2002a). Our laboratory also used deep sequencing of small RNAs to reveal the molecular regulatory network of *AtENO2* on the seed germination rate (Wu et al., 2021). However, the role of *AtENO2* in germination under salt stress is unclear. We found that *eno2^-^* was hypersensitive to NaCl and mannitol treatments during seed germination but less sensitive to KCl treatment compared to the WT (**Figure 1**), which revealed that *AtENO2* was involved in seed germination by affecting the content of Na+ and osmotic pressure. Together, these results clearly showed that *AtENO2* responded to multiple abiotic stresses and that *AtENO2* was a positive germination regulator under salt stress.

### *AtENO2* regulates a large array of stress-responsive genes and corresponding proteins

Integrative transcriptomic and TMT-based proteomic analysis revealed many DEGs and DEPs involved in the response to stimulus, and stresses were enriched in *eno2^-^* compared to the WT under salt stress (**Figure 4D**). The expression of a large number of stress-responsive genes and corresponding proteins was significantly lower in the mutant. Water transportation is a major physiological process of vegetative and reproductive growth in plants that can be tightly influenced under different conditions (Blum, 2009; Wang et al., 2021). Aquaporins (AQPs) play a central role in the symplastic pathway, which is efficient in transporting water across membranes (Maurel et al., 2008; Maurel et al., 2015). In addition to transporting water, AQPs also involve many physiological and developmental processes, including seed germination, reproductive growth, and stress responses in plants (Durbak et al., 2014; Beamer et al., 2021; Su et al., 2022). *PIP1B* (*PIP1;2*) is a vital member of the *Arabidopsis* aquaporin family. Essential for the water permeability of the plasma membrane and the morphology of the root system (Tournaire-Roux et al., 2003). Under normal conditions, there was no significant difference in the expression of PIP1B between the WT and *eno2^-^*, but it was significantly reduced under salt stress. The trend of gene expression was the same as that of protein expression (**Figure 5**). We hypothesized that under salt stress, the expression of PIP1B in *eno2^-^* was significantly reduced, and the water transport capacity was also lower, thus affecting the seed germination process.

The activity of peroxidases is positively correlated with plant resistance (Gaudet et al., 2011); that is, the higher its activity is, the stronger the stress resistance of the plant. Hydrogen peroxide (H_2_O_2_) is one of the important representatives of ROS (Liu et al., 2018; Wu et al., 2020). Hormone signals and abiotic stress can induce the production and accumulation of H_2_O_2_ in plant cells. Under normal conditions, the production and removal process of H_2_O_2_ maintains a fine dynamic balance, which not only ensures the physiological function of H_2_O_2_ in plants but also minimizes the harmful effect on plants. If the balance of H_2_O_2_ production and scavenging is broken, excessive accumulation of H_2_O_2_ occurs, which can cause oxidative damage due to its high redox activity. To reduce the toxicity of H_2_O_2_, plants have formed complex and effective coping mechanisms, mainly including superoxide dismutase (SOD), catalase (CAT), ascorbate peroxidase (APX), and peroxidase (POD) enzymatic scavenging systems, as well as ascorbic acid, oxidized glutathione, and other nonenzymatic scavenging systems that can remove H_2_O_2_ (Akbar et al., 2020; Meng et al., 2022). APXT plays a key role in hydrogen peroxide removal. APX4 was originally thought to be an ascorbate peroxidase (Panchuk et al., 2002; Lu et al., 2007). Under normal growth conditions, the expression of APXT and APX4 in *eno2^-^* was lower than that in the WT, and the difference was more obvious under salt stress (**Figure 5**). All these results revealed that more H_2_O_2_ accumulated in the mutant, and the balance was broken, which affected the germination process of seeds under salt stress.

### *AtENO2* affects phytohormone-related genes and corresponding proteins

In addition to external stress-related factors, internal phytohormones also play an indispensable role in seed germination. As an important regulatory factor of plant growth, ABA can promote seed dormancy, inhibit seed germination, inhibit seedling root growth, promote leaf senescence, and participate in a variety of metabolic synthesis pathways and signal transduction pathways (Breeze, 2020; Varshney and Majee, 2021; Ali et al., 2022; Mphande et al., 2022). GA and ABA play opposite roles in seed germination. GA can break seed dormancy and promote seed germination (Liu and Hou, 2018; Abley et al., 2021). Seeds of GA-deficient mutants *ga1* and *ga2* showed an enhanced dormancy phenotype, while exogenous GA could promote seed germination (Lee et al., 2002b; Shu et al., 2013). In the absence of GA, DELLA proteins such as RGL2, a negative regulator, promote the expression of XERICO, a ring finger protein that encodes ABA synthesis, which in turn increases the expression of RGL2 and ABA-signaling proteins such as ABI5, thereby inhibiting seed germination. GA interrupts this pathway by promoting the degradation of DELLA, which promotes the germination process of seeds (Piskurewicz et al., 2008; Ariizumi et al., 2013; Park et al., 2013).

In our research, integrative transcriptomic and proteomic analyses revealed that a large number of DEGs and DEPs involved in the response to the hormones were enriched in *eno2^-^* compared to the WT under salt stress (**Figure 4D**). After *ENO2* deletion, the expression levels of many genes and proteins related to phytohormone synthesis were significantly changed. 3-Ketoacyl-CoA thiolase-2 (PED1/KAT2/PKT3) (EC 2.3.1.16) is an enzyme catalyzing the β-oxidation of fatty acids involved in ABA signaling. Previous research has proven that PED1 positively regulates ABA signaling in all major ABA responses, including ABA-induced inhibition of seed germination and postgermination growth arrest and ABA-induced stomatal closure and stomatal opening inhibition in *Arabidopsis thaliana* (Jiang et al., 2011). Our results showed that the content of PED1 was prominently upregulated in *eno2^-^* under salt stress, which indicated that the concentration of ABA also increased correspondingly. We noticed that the protein expression indices of HY5 and ACO3 related to ABA were also upregulated in *eno2^-^* compared to the WT under salt stress. In addition, we found a protein named GASA1, which may function in hormonal controlled steps of development such as seed germination, flowering, and seed maturation and was downregulated notably in *eno2^-^* under salt stress. Interestingly, GASA1 was also downregulated by ABA (Raventos et al., 2000). Together, the change in these phytohormones corresponded with the lower seed germination rate in the *eno2^-^* mutant.

### AtENO2 interacts with GAPA1/GAPB to regulate seed germination under salt stress

Interaction with other proteins is one of the important mechanisms of protein functions. Protein interaction experiments confirmed that AtENO2 could interact with GAPA1 and GAPB (**Figure 7**). Whether it was normal or under salt stress, the GAPA1/GAPB content in *eno2^-^* was markedly lower than that in the WT (**Figure 5**). GAPDH is a constitutively expressed enzyme that plays a vital role in carbon metabolism mainly by participating in glycolysis. There are four major GAPDHs in higher plants: GAPA/GAPB, GAPC, NP-GAPDH, and GAPCp. The expression of GAPDH is very abundant in organisms, and it is generally expressed constantly in the same tissues and is highly conserved; therefore, it is usually used as a housekeeping gene. However, an increasing number of studies have shown that GAPDH not only participates in glycolysis and the Calvin cycle but also plays an important role in protein transport and modification, cell apoptosis control, and response to abiotic stress (Sirover, 2018; Dar et al., 2021; Lim et al., 2021). *GAPC* can respond to a variety of stresses and can play an indispensable regulatory role in the process of plant resistance to stress (Zhang et al., 2019; Yang et al., 2021b). The main reason for this may be that as the main regulator of energy metabolism in cells, plants can maintain their growth under stress by adjusting the energy and metabolism in cells. GAPA and GAPB, which we focused on, are NADPH-specific phosphorylases that play a role in carbon dioxide fixation in chloroplast photosynthesis. Previous studies have shown that overexpressing GAPB in *Arabidopsis thaliana* can improve its salt resistance (Chang et al., 2015), which may be because GAPB can maintain the high circulation of ADP and NADP+ under salt stress, thus reducing the damage caused by excessive ROS production and maintaining the photosynthetic efficiency and growth of plants under high salt stress. Under salt stress, the contents of GAPB and GAPA1 in *eno2^-^* decreased significantly, indicating that their corresponding photosynthetic efficiency also decreased notably. Correspondingly, we detected chlorophyll content in seedlings of different lines growing for the seventh day, and the chlorophyll content in the mutant was prominently lower than that in the WT (**Supplementary Figure S2**). Therefore, we inferred that ENO2 could regulate seed germination by forming the ENO2-GAPA1 or ENO2-GAPB complex under salt stress in *Arabidopsis thaliana.* Of course, the role of GAPA1 or GAPB in seed germination still needs to be further studied.

## Materials and methods

### Plant materials and growth conditions

The ecotype Columbia (Col-0) of *Arabidopsis thaliana* was used as the WT, and the *AtENO2* T-DNA insertion mutants *los2-2* (also called *eno2^-^*) (SALK_021737), *los2-3* (SALK_077784), and *los2-4* (SAIL_208_B09) were obtained from the Arabidopsis Biological Resource Center (ABRC) at Ohio State University. The details of these mutants were described previously (Eremina et al., 2015). Because *los2-3* and *los2-4* were embryonic lethal, we only used *eno2^-^* in our research. The *eno2^-^*/35S:*AtENO2* lines (*ER*, the CDS of entire *AtLOS2* rescued to *eno2^-^*) were preserved in our lab. Seeds were surface-sterilized for 5 min in 2% sodium hypochlorite (Lvtao Environ-mental Technology, Cangzhou, China) and rinsed three times with sterile water. The seeds were plated on 1/2 MS medium supplemented with 1.5% sucrose (w/v) (Coolaber, Beijing, China) and 0.8% agar (w/v) (Coolaber, Beijing, China). The plates were placed at 4°C for 3 days and moved to a long-day (16 h light/8 h dark) growth room at 21°C for subsequent research.

### Seed germination assay

To explore the role of *AtENO2* in the stress response, the WT, *eno2^-^*, and *ER* (the CDS of entire *AtLOS2* rescued to *eno2^-^*) constructed, screened, and identified in our laboratory (Liu et al., 2020) were used as materials to calculate germination status. The seeds of the WT, *eno2^-^*, and *ER* were germinated on 1/2 MS medium with or without 100 mM NaCl, isotonic mannitol, or 100 mM KCl (the same concentration as NaCl), and the germination rates were checked every day for 7 days. We regarded radicle breaks through seed coats as germination (Zhao et al., 2021a). The germination and cotyledon greening of each line were counted at a fixed time every day. The average seed germination rate was obtained from three experimental replicates, and approximately 30 seeds were observed for each genotype. The seed germination rate was obtained by counting the number of germinated seeds divided by the total number of seeds tested. On the seventh day, the overall situation of germination and leaves were photographed, and the width and length of leaves were measured for further statistical analysis. Meanwhile, we collected and immediately froze the seedlings on the seventh day in liquid nitrogen. The samples were stored at -80°C until RNA and protein extraction.

### Chlorophyll content measurement

Chlorophyll was extracted from seedlings germinated for seven days using 95% ethanol (v/v) and the absorbance of the supernatant was measured using a SpectraMax Plus 384 (Molecular Devices, USA). Chlorophyll content was estimated as described by the following equation (Wintermans and Demots, 1965).

### Transcriptome profiling

Total RNA was extracted from the seedlings (WT and *eno2^-^*) on the seventh day under normal and salt conditions (100 mM) using TRIzol^®^ Reagent according to the instructions (Magen, Guangzhou, China). RNA samples were detected based on the A260/A280 absorbance ratio with a Nanodrop ND-2000 system (ThermoFisher Scientific, Waltham, USA), and the RIN of RNA was determined by an Agilent Bioanalyzer 4150 system (Agilent Technologies, California, USA). Only qualified samples were used for library construction. Paired-end libraries were prepared using an ABclonal mRNA-seq Lib Prep Kit (ABclonal, Wuhan, China) following the manufacturer’s recommendations. mRNA was enriched by oligo (DT) beads and decomposed by fragmentation buffer. These short fragments were reverse-transcribed into cDNA using random primers, and the second-strand cDNA was subsequently synthesized using dNTPs, DNA polymerase I, RNase H, and buffer. The ligated products were selected by agarose gel electrophoresis, PCR amplified, and then sequenced on an Illumina Novaseq 6000/MGISEQ-T7 instrument.

The data generated from the Illumina/BGI platform were used for bioinformatics analysis. Clean data were obtained by removing unqualified reads with ambiguous nucleotides, and adapter sequences were filtered from raw data. Then, the clean data were separately aligned to the reference genome with orientation mode using HISAT2 software (http://daehwankimlab.github.io/hisat2/) to obtain mapped reads. The mapped reads were spliced with StringTie software (http://ccb.jhu.edu/software/stringtie/), and we used Gffcompare software (http://ccb.jhu.edu/software/stringtie/gffcompare.shtml) to compare them with the reference genome GTF/GFF file to detect the original unannotated transcription region and find new transcripts and new genes of the species. We used FeatureCounts (http://subread.sourceforge.net/) to count the read numbers that mapped to each gene. Then, the FPKM of each gene was calculated based on the length of the gene, and the read count was mapped to this gene. DESeq2 was used to screen the DEGs, and DEGs with |log2FC|>1, and p value<0.05 were considered to be significant DEGs. The volcano plot and UpSet graph were drawn with the help of the Omicstudio cloud platform (https://www.omicstudio.cn/tool/43). The GO was plotted by the OmicShare cloud platform (https://www.omicshare.com/).

### Protein extraction, protein digestion, and TMT labeling

Total proteins were extracted from seedlings (WT and *eno2^-^*) on the seventh day under normal and salt conditions (100 mM) using SDT buffer (4% (w/v) SDS, 100 mM Tris/HCl, 1 mM DTT, pH 7.6), and the amount of protein was quantified with the BCA Protein Assay Kit (Bio–Rad, California, USA). Protein digestion by trypsin was performed according to the filter-aided sample preparation (FASP) procedure described by Jacek R Wiśniewski (Wisniewski et al., 2009). The digest peptides of every sample were desalted on C18 Cartridges (Empore™ SPE Cartridges C18 (standard density), bed I.D. 7 mm, volume 3 ml, Sigma), concentrated by vacuum centrifugation and reconstituted in 40 µl of 0.1% (v/v) formic acid. We took 100 μg of peptide from each sample and labeled it according to the instructions of the kit (ThermoFisher Scientific, Waltham, USA). The samples were labelled (WT-1)-126, (WT-2)-127 N, (WT-3)-127 C, (*eno2–*1)-128 N, (*eno2–*2)-128 C, (*eno2–*3)-129 N, (WT-S-1)-129 C, (WT-S-2)-130 N, (WT-S-3)-130 C, (*eno2^-^*--S-1)-131 N, (*eno2^-^*--S-2)-131 C, and (*eno2–*S-3)-132 N. Our project designed 4 groups, and each group contained 3 biological repeat samples, for a total of 12 samples.

### High pH reversed-phase fractionation and liquid chromatography-tandem mass spectrometry analysis

The labeled peptides in each group were mixed in equal amounts, and the labeled peptides were fractionated by the High pH Reversed-Phase Peptide Fractionation Kit (ThermoFisher Scientific, Waltham, USA). The dried peptide mixture was reconstituted and acidified with 0.1% TFA solution and loaded onto an equilibrated, high-pH, reversed-phase fractionation spin column. The peptide segments were combined with hydrophobic resin in the aqueous phase and desalted by low-speed centrifugation and washing the column with water. A step gradient of acetonitrile concentration in a volatile high pH elution solution was then applied to the column to elute the binding peptide into 10 distinct parts by centrifugal collection. The collected fraction was desalted on C18 cartridges (Empore™ SPE Cartridges C18 (standard density), bed I.D. 7 mm, volume 3 ml) and then concentrated by vacuum centrifugation.

Each sample in the project was separated by an Easy nLC HPLC system at a nanolitre flow rate. Buffer Solution A was a 0.1% formic acid aqueous solution, and buffer Solution B was a 0.1% formic acid acetonitrile aqueous solution (acetonitrile was 84%). The chromatographic column was balanced with 95% Liquid A, and the samples were transferred from an automatic sampler to the loading column and separated by an analytical column at a flow rate of 300 NL/min. The chromatographic column was balanced with 95% Liquid A, and the samples were transferred from an automatic sampler to the loading column (Thermo Scientific Acclaim PepMap100, 100 μm*2 cm, nanoViper C18) and separated by an analytical column (Thermo Scientific Easy Column, 10 cm long, 75 μm inner diameter, 3 μm resin) at a flow rate of 300 NL/min. After chromatographic separation, the samples were analyzed by a Q-exactive mass spectrometer (ThermoFisher Scientific, Waltham, USA). The detection method was positive ion, the scanning range of parent ion was 300-1800 m/z, the resolution of primary mass spectrometry was 70000 at 200 m/z, the target of automatic gain control (AGC) was 1e6, the maximum was 50 ms, and the dynamic exclusion time was 60.0 s. The mass charge ratios of peptides and peptide fragments were collected as follows: Twenty MS2 Scan were collected after each full scan. The MS2 activation type was HCD, and the isolation window was 2 m/z. The secondary mass spectrometry resolution was 17,500 at 200 m/z. The normalized collision energy was 30 eV, and the underfill was 0.1%.

### Bioinformatics analysis of proteomic data

The MS raw data for each sample were searched using the MASCOT engine (Matrix Science, London, UK; Version 2.2) embedded into Proteome Discoverer 1.4 software for identification and quantitation analysis. The volcano plot and UpSet graph were drawn with the help of the Omicstudio cloud platform (https://www.omicstudio.cn/tool/43). The GO was plotted by the OmicShare cloud platform (https://www.omicshare.com/). KEGG and PPI analyses were performed using OmicsBean (http://www.omicsbean.cn/dashboard/).

### RNA extraction and qRT–PCR assay

Total RNA was extracted from seedlings on the seventh day of germination using Eastep^®^ Super (Promega, Madison, USA). Total RNA from each sample was converted into cDNA by using First-Strand cDNA Synthesis SuperMix (TransGen, Beijing, China). The specific forward primers and the universal reverse primer were designed by Primer 5.0 (**Table S11**). qRT**–**PCR was performed using TransStart^®^ Top Green qPCR SuperMix (TransGen, Beijing, China) on an ABI7500 Real-time PCR system. The 20 μL reactions contained 2 μL reverse transcription cDNA products, 10 μL qPCR SuperMix, 2 μL primer mix, and supplemented with nuclease-free water to 20 μL. The qRT-PCR were performed as follows: 94°C for 30 s, followed by 40 cycles of 94°C for 5 s and 60°C for 30 s. Each reaction had three technical replicates, and the expression levels were calculated using the 2^−ΔΔCt^ method. *UBQ5* was used as the internal standard.

### Luciferase complementation assay

The coding sequences of AtENO2 were cloned into pCAMBIA1300-nLuc, while the coding sequences of AtGAPA1 and AtGAPB were cloned into pCAMBIA1300-cLuc. The sequences of primers for these constructs are listed in **Table S12**. The pairs of Agrobacterium tumefaciens strain (GV3101) carrying ENO2-nLuc/cLuc-GAPA1, ENO2-nLuc/cLuc, nLuc/cLuc-GAPA1, ENO2-nLuc/cLuc-GAPB, nLuc/cLuc-GAPB, and nLuc/cLuc were cotransformed into four-week N. *benthamiana* leaves. After infiltration, plants were covered with plastic bags and placed at 21°C for 24 h in a dark room. Plants were then incubated at 21°C with 48 h light before the signals were detected with CCD (Chen et al., 2008).

### Statistical analysis

The data are presented as the means ± SD and were compared using SPSS software with one-way ANOVA followed by Duncan’s multiple range test at a significance level of p < 0.05. Different letters represent a significant difference.

### Accession numbers

The sequence information in our study could be found in the National Center for Biotechnology Information (NCBI) under the following accession numbers: *ENO2* (*LOS2*) (At2g36530), *ENO1* (At1g74030), *ENO3* (At2g29560), *UBQ5* (At3g62250), *AUX1* (AT2G38120), *PIN4* (AT2G01420), *AT3G22850* (AT3G22850), *ACO3* (AT2G05710), *HY5* (AT5G11260), *LHCB3* (AT5G54270), *PED1* (AT2G33150), *GASA1* (AT1G75750), *AOC2* (AT3G25770), *GAPA1* (AT3G26650), *GAPB* (AT1G42970), *APXT* (AT1G77490), *PIP1B* (AT2G45960), *CRY1* (AT4G08920), *APX4* (AT4G09010), *GGT1* (AT4G39640), *PGK1* (At3g12780).

## Conclusion

**Figure 8:**
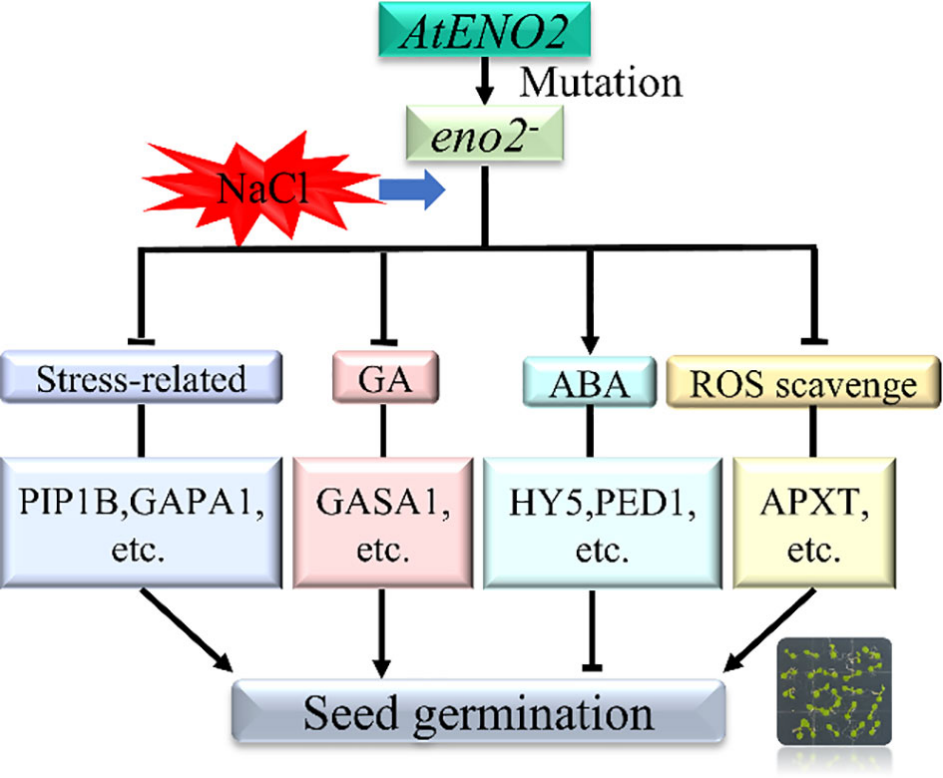
Mechanistic model of *AtENO2* affecting seed germination under salt stress Under salt stress, *AtENO2* gene mutation up-regulated genes and proteins related to the ABA signaling pathway. The mutant also down-regulated genes and proteins related to stress, GA, and ROS scavenging, ultimately affecting seed germination rate.

This is the first systematic report on the mechanism by which *AtENO2* (*AtLOS2*) affects seed germination under salt stress in *Arabidopsis thaliana* based on transcriptome and proteomic analysis. Phenotypic observation and statistics revealed that *AtENO2* acts as a positive regulator in the stress response at the germination stage. The combined analysis found many DEGs and DEPs in *eno2^-^* under salt stress compared to the WT. Genes and corresponding proteins related to stresses (PIP1B, GAPA1, GAPB, etc.) were highly downregulated in the mutant, and the DEGs and DEPs related to hydrogen peroxide removal (APXT, APX4, etc.) were also downregulated in *eno2^-^*. In addition, several DEGs and DEPs encoding phytohormone transduction pathways, i.e., auxin (PIN4, and AUX1) and GA (GASA1) were identified and may have a vital role in the contribution to salt tolerance in seed germination under *AtENO2* deficiency. The DEGs or DEPs related to ABA signalings, such as PED1, ACO3, and HY5, were relatively highly upregulated in *eno2^-^* under salt stress. These genes or proteins could participate in salt sensitivity and seed dormancy. Moreover, we constructed an interactive network with OmicsBean and further identified GAPA1 and GAPB that could interact with AtENO2 by LCA to explain the function of AtENO2 under salt stress during seed germination. Overall, all the results reveal that under salt stress, *AtENO2* mainly affects the expression of genes and proteins related to the phytohormone signal transduction pathways, stress response factors, and ROS, and then affects seed germination (**Figure 8**). Our research provides a better understanding of the molecular function of *AtENO2* in enhancing salt resistance in *Arabidopsis thaliana* at the seed germination stage.

## Data availability statement

The RNA-Seq data presented in the study are deposited in the NCBI, PRJNA839519. The proteomics data have been deposited to ProteomeXchange Consortium via the iProX partner repository, PXD033483.

## Author Contributions

GZ and YW designed this project. YW performed most of the experiments and analyzed the data. YW drew all the graphs, and YW wrote the paper. HL assisted in the experiment of seed germination. JB collected some references related to omics. GZ provided financial support. All authors contributed to the article and approved the submitted version.

## Funding

This study was funded by the National Natural Science Foundation of China (31872672) and the Open fund from the Beijing Key Laboratory of Gene Resource and Molecular Development.

## Acknowledgments

We would like to thank the experimental technology center for life sciences, Beijing Normal University. We are also grateful to Shanghai Applied Protein Technology for assisting in sequencing.

## Conflict of interest

The authors declare that the research was conducted in the absence of any commercial or financial relationships that could be construed as a potential conflict of interest.

## Publisher’s note

All claims expressed in this article are solely those of the authors and do not necessarily represent those of their affiliated organizations, or those of the publisher, the editors and the reviewers. Any product that may be evaluated in this article, or claim that may be made by its manufacturer, is not guaranteed or endorsed by the publisher.
