# Supplementary material for: Integrative transcriptomic and TMT-based proteomic analysis reveals the mechanism by which AtENO2 affects seed germination under salt stress
Source: Front Plant Sci. 2022 Oct 21;13:1035750. doi: 10.3389/fpls.2022.1035750 (PMC9634073; doi:10.3389/fpls.2022.1035750)
Supplement: Supplementary file 1 [file DataSheet_1.docx]

**Figure S1**

**
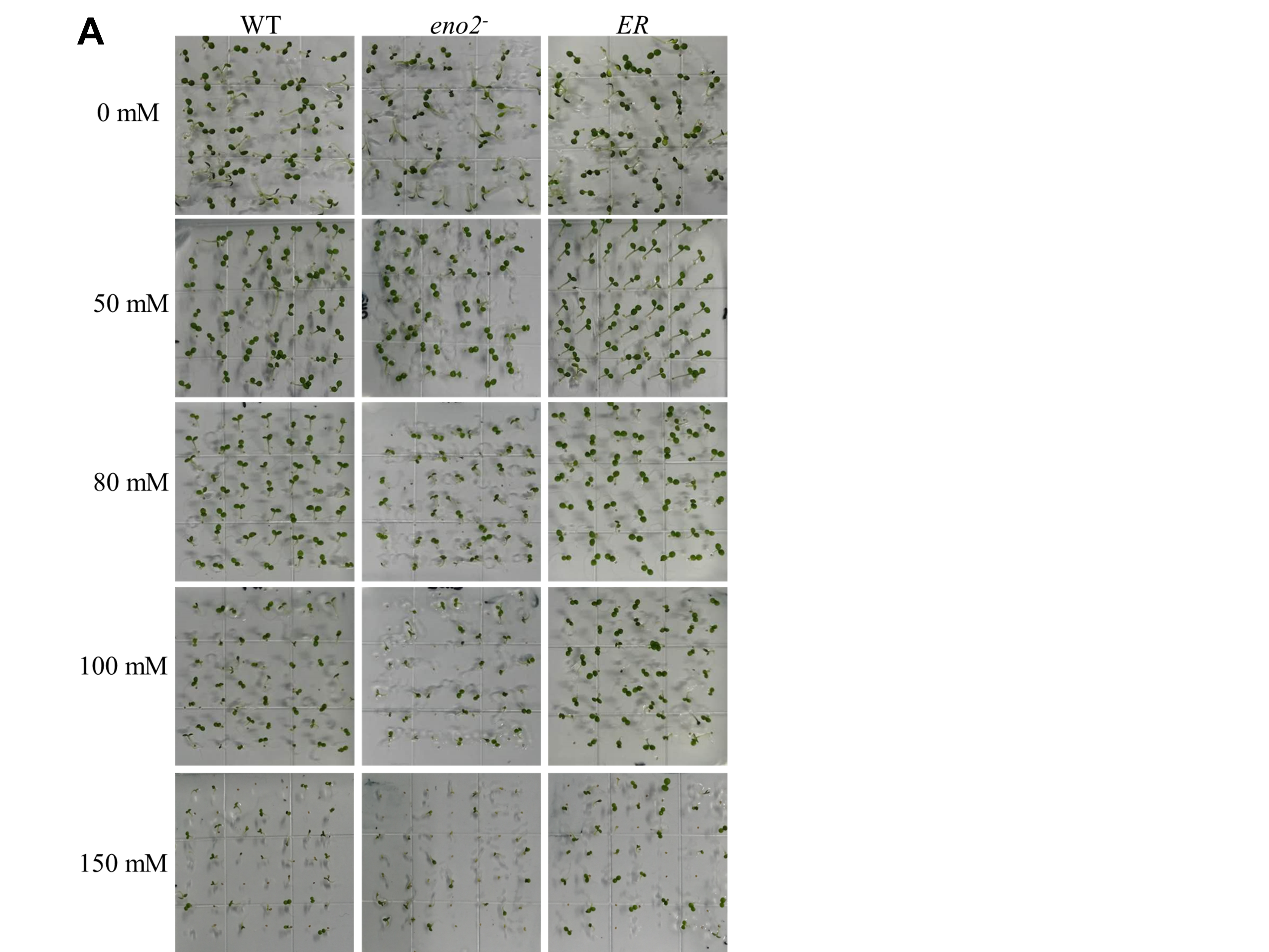
**

**
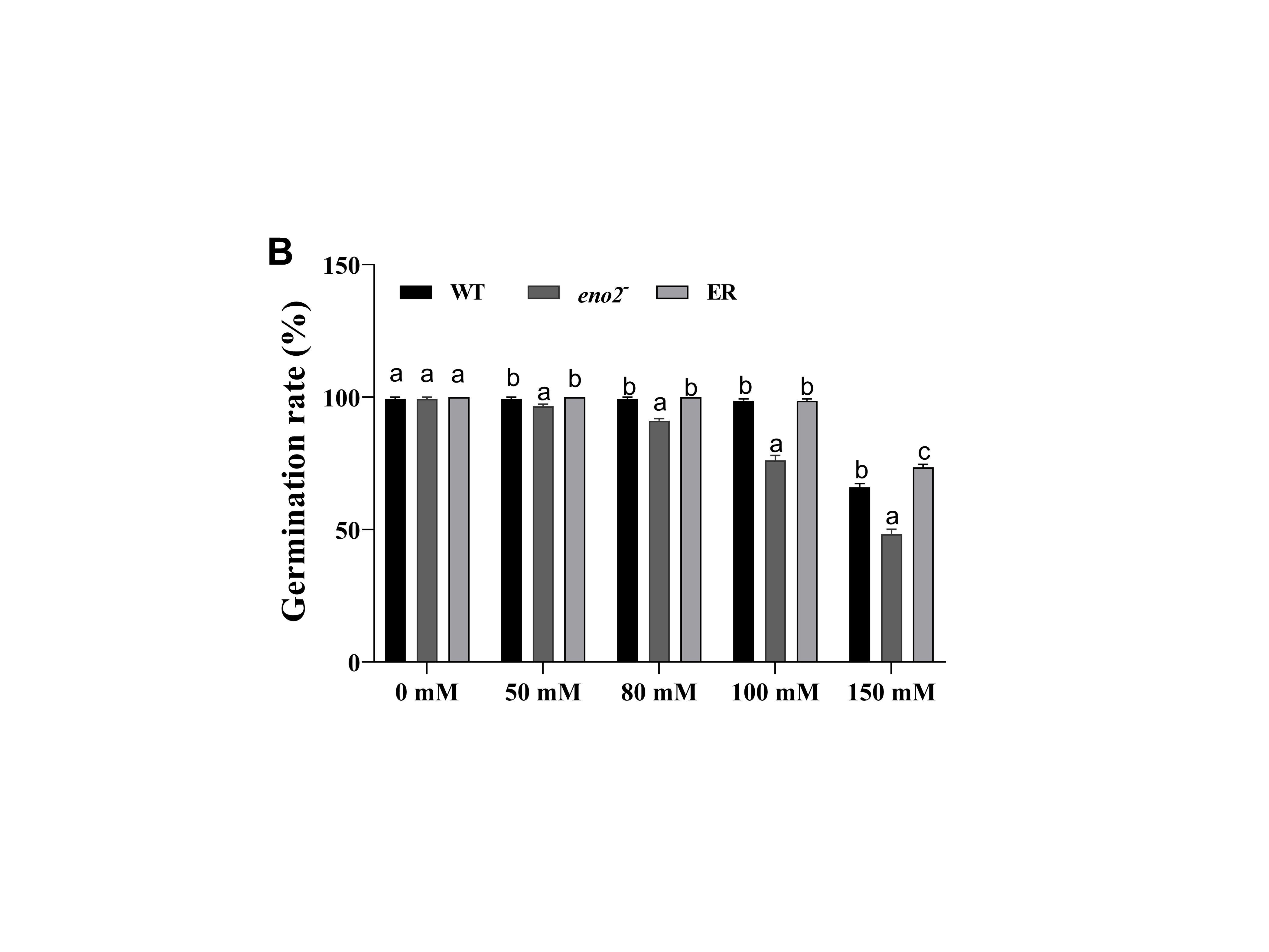
**

Figure S1 Germination of each line under different salt concentrations.

Seed germination of the WT, *eno2^-^*, and ER lines. Seeds were horizontally germinated on 1/2 MS medium with different concentrations of NaCl for 7 days. Photographs were taken on the seventh day of germination (A). The seed germination rate was measured on the seventh day (B). Values are expressed as the means ± SD (n=3 replicates). Different letters indicate significant differences by one-way ANOVA (P<0.05) with SPSS.

**Figure S2**

**
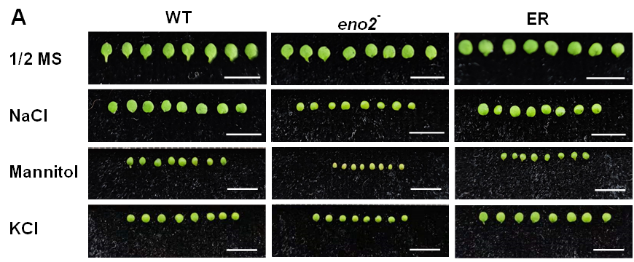
**

**
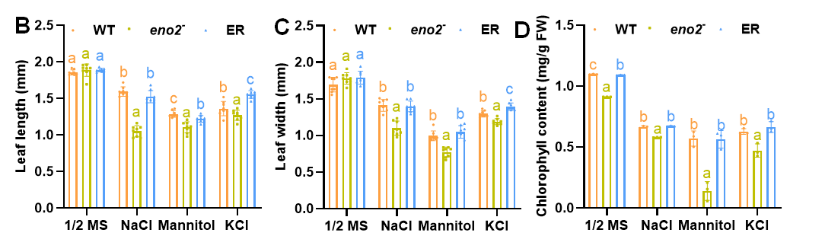
**

Figure S2 *AtENO2* regulates seedling leaf size and chlorophyll content under abiotic stress.

Leaf size and chlorophyll content of the WT, *eno2^-^*, and ER lines. Seeds were horizontally germinated on 1/2 MS medium with or without 100 mM NaCl, 200 mM mannitol, and 100 mM KCl for 7 days. Photographs were taken on the seventh day of seedling leaf development (A). The leaf length was measured on the seventh day (B). The leaf width was measured on the seventh day (C). The chlorophyll content in seedlings on Day 7 was measured (D). The bar=0.5 cm. The orange, green, and blue histograms represent the WT, *eno2^-^*, and ER, respectively. Values are expressed as the means ± SD (n=3 replicates). Different letters indicate significant differences by one-way ANOVA (P<0.05) with SPSS.

**Figure S3**


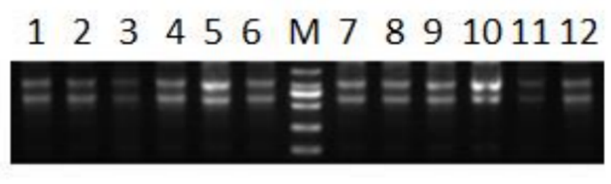


Figure S3 Agarose gel electrophoresis of the total RNA.

1,2,3, represent the untreated samples with 3 replicates of WT seedlings; 4,5,6, represent the untreated samples with 3 replicates of *eno2^-^* seedlings; 7,8,9, represent the treated samples (100 mM NaCl) with 3 replicates of WT seedlings; 10,11,12, represent the treated samples (100 mM NaCl) with 3 replicates of *eno2^-^* seedlings.

**Figure S4**


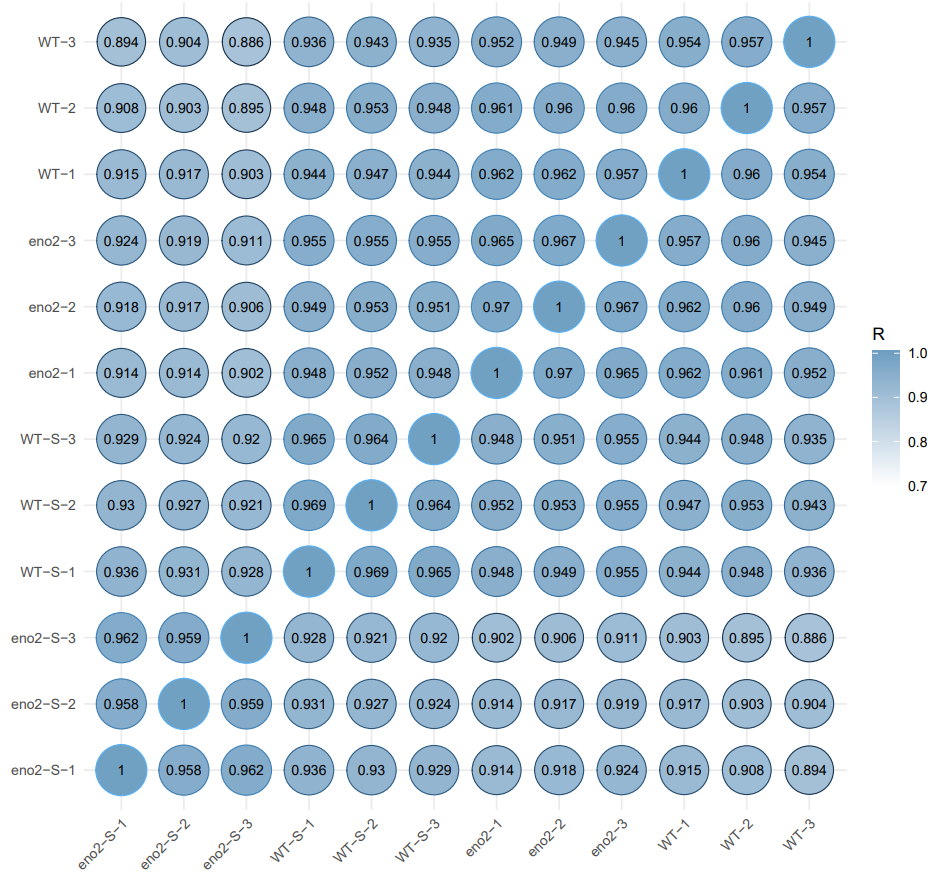


Figure S4 Heatmap of the correlation coefficient between samples

The X-axis and Y-axis in the figure represent different samples. The number in the circle represents the correlation coefficient of the two samples. The darker the circle is, the higher the correlation is. R represents the Pearson correlation coefficient, and the R values between biological samples were required to be greater than 0.8.

**Figure S5**


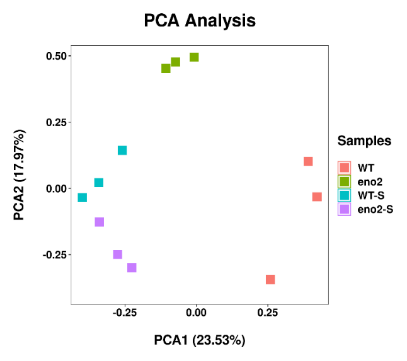


Figure S5 Principal component analysis (PCA) of transcriptome samples

The X-axis is the first principal component and the Y-axis is the second principal component. The points with the same color in the figure represent each biological repetition in the group, and the distance between points represents the overall expression difference of the sample.

**Figure S6**


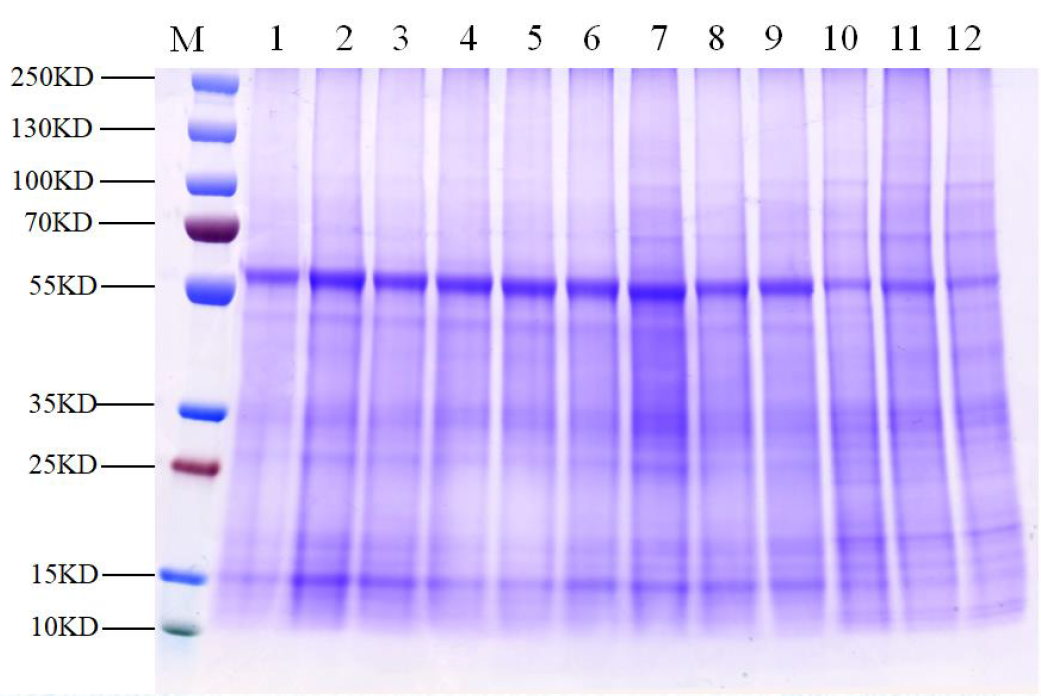


Figure S6 Protein SDS-PAGE electrophoretogram

1,2,3, represent the untreated samples with 3 replicates of WT seedlings; 4,5,6, represent the untreated samples with 3 replicates of *eno2^-^* seedlings; 7,8,9, represent the treated samples (100 mM NaCl) with 3 replicates of WT seedlings; 10,11,12, represent the treated samples (100 mM NaCl) with 3 replicates of *eno2^-^* seedlings.

**Figure S7**


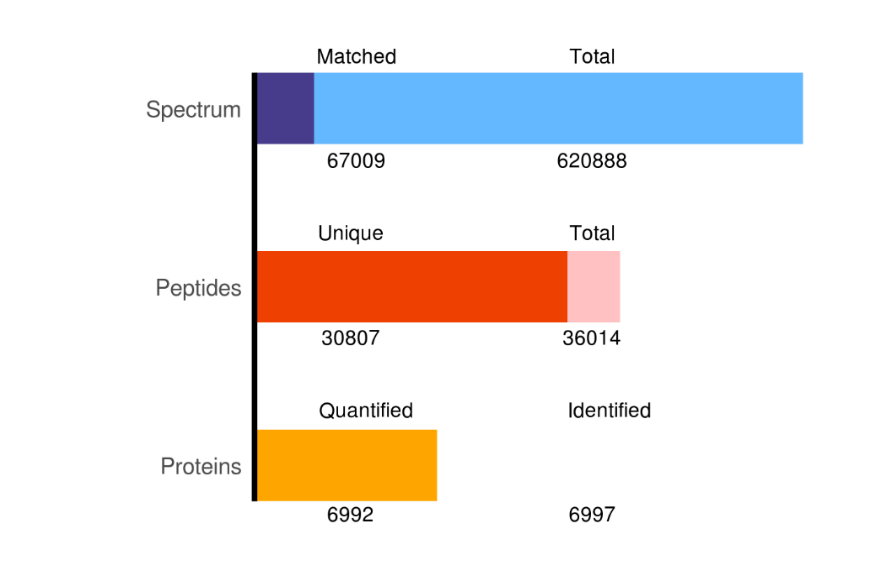


Figure S7 Statistical histogram of identification and quantification results

Quantifiable protein means in at least one comparison group, more than half of the biological repeats have the strength value of the protein.

**Figure S8**


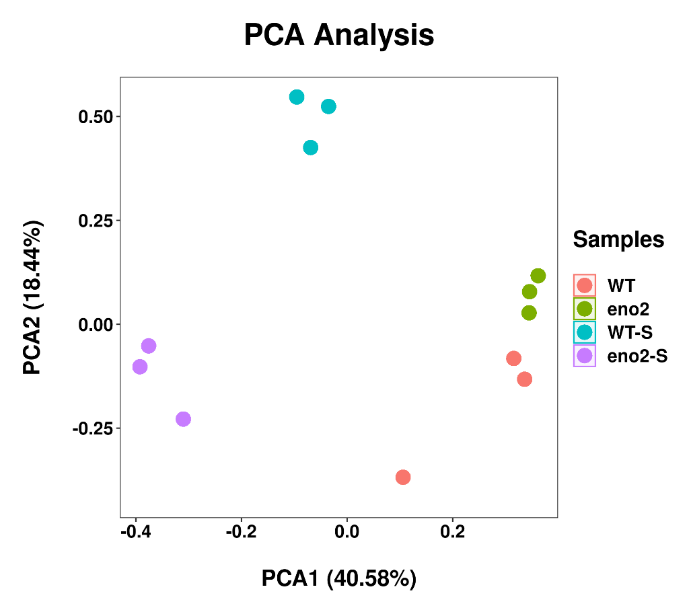


Figure S8 Principal component analysis of proteomic samples

The X-axis is the first principal component and the Y-axis is the second principal component. The points with the same color in the figure represent each biological repetition in the group, and the distance between points represents the overall expression difference of the sample.
